# Fundamentally unchanged northwestern African rainfall regimes across the Plio-Pleistocene transition

**DOI:** 10.1126/sciadv.ads3149

**Published:** 2025-06-20

**Authors:** Bryce A. Mitsunaga, Amy M. Jewell, Solana Buchanan, Anya J. Crocker, Paul A. Wilson, Timothy D. Herbert, James M. Russell

**Affiliations:** ^1^ Department of Earth, Environmental, and Planetary Sciences, Brown University, Providence, RI 02912, USA.; ^2^ University of Southampton, Waterfront Campus, National Oceanography Centre, Southampton, SO14 3ZH, UK.; ^3^ Earth, Environmental, and Planetary Sciences, Rice University, Houston, TX 77005, USA.

## Abstract

Northern African climate is characterized by strongly contrasting wet summers and dry winters. Dust exported by the northeasterly trade (Harmattan) winds creates marine sedimentary records that have been long interpreted to show that northern African climate became drier and more variable across the Pliocene-Pleistocene boundary [2.58 million years ago (Ma)], when global climate cooled and high-latitude glacial-interglacial cycles intensified. However, questions about the impact of summer rainfall on winter dust fluxes and thus the history of the African summer monsoons remain. We present a leaf wax hydrogen isotope record from offshore northwestern Africa that demonstrates that rainfall regimes remained stable and varied solely in response to 21,000-year cycles in summer insolation from 3.5 to 2.5 Ma. We infer that the summer rains and winter winds respond to different climate forcings, with summer rainfall driven by solar radiation over the northern African landmass and the winter trades affected by high-latitude climate and meridional temperature gradients.

## Teaser

The northern African summer monsoon was unaffected by global cooling at the end of the Pliocene 3 million years ago.

## INTRODUCTION

The Sahara and Sahel undergo extreme wet-dry and desert-grassland cycles on seasonal to orbital timescales (*1*–*3*). The region is prone to high levels of water stress (*4*), and poor agreement among future rainfall projections challenges climate change adaptation and mitigation efforts (*5*, *6*). Long-term hydroclimate reconstructions of past warm climates can provide useful context to disentangle the impacts of different forcings and feedbacks on the African monsoons. Although data constraining recent northern African climate variations have improved (*7*), long, high-resolution records of the highly seasonal (summer) monsoonal rainfall are still rare.

**Fig. 1.**
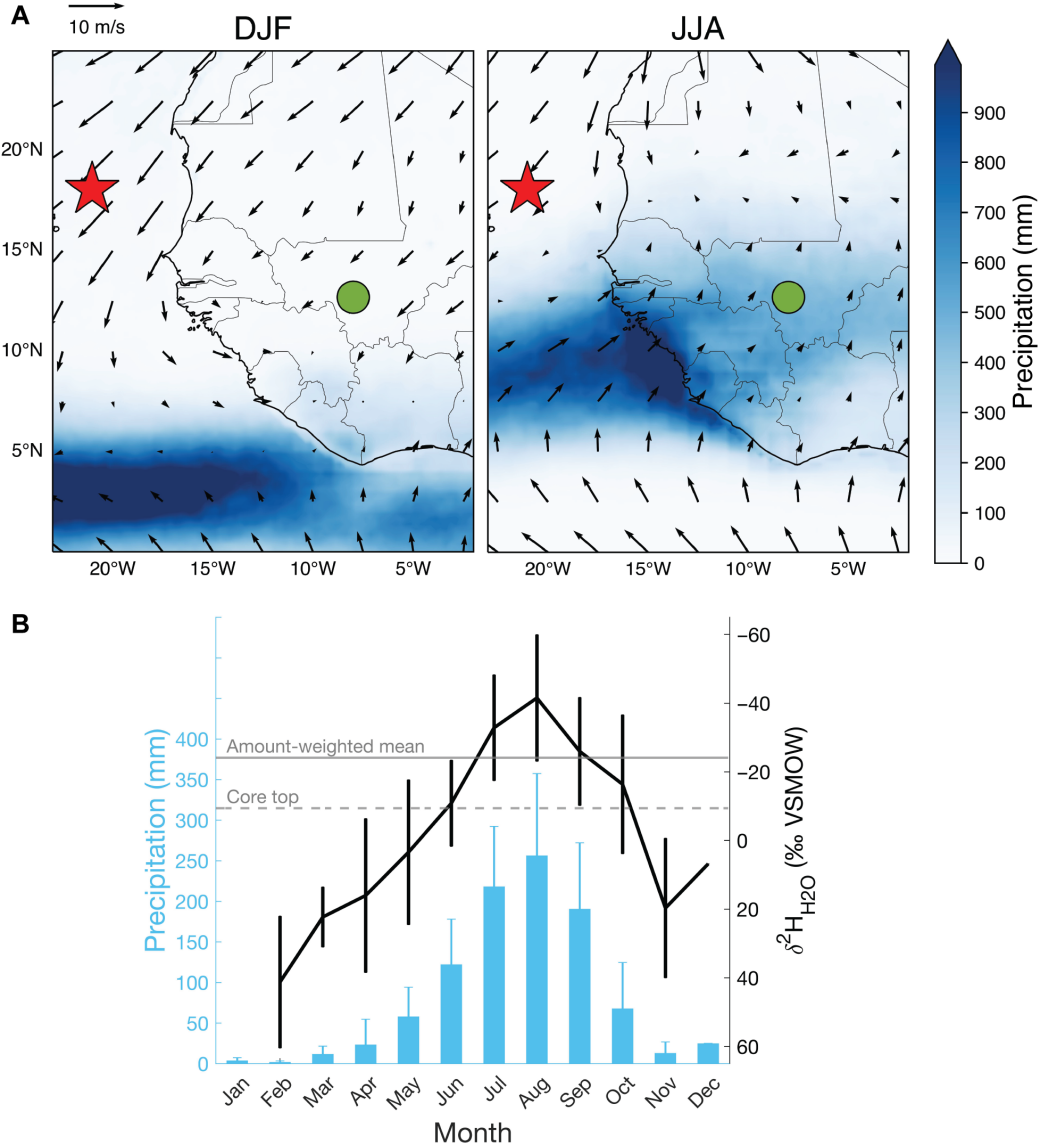
Modern northwestern African seasonal hydroclimatology. (**A**) 1998–2015 mean December–February (DJF) and June–August (JJA) precipitation (*92*), 2020 surface level wind vectors (*93*), and locations of ODP Site 659/MD03-2705 (red star) and Bamako GNIP station (green circle) (*57*). (**B**) Bamako 1962–2018 monthly mean precipitation amounts and δ^2^H_H2O_ values with the mean annual δ^2^H_H2O_ average and core top δ^2^H_wax_- and δ^13^C_wax_-inferred δ^2^H_H2O_ values. VSMOW, Vienna standard mean ocean water.

Ocean Drilling Program (ODP) Site 659 (18.08°N, 21.03°W, 3070-m water depth; Fig. 1A) has provided an archive of northwestern African climate spanning the Plio-Pleistocene [5 to 0 million years ago (Ma)], which includes global cooling and intensification of Northern Hemisphere glaciation (iNHG) from ~2.73 Ma (*8*), strengthening of 41,000–thousand year (kyr) glacial-interglacial cycles (2.8 to 2.5 Ma) (*9*), an increase in global and regional (Saharan) windiness (*10*, *11*), and enhanced pole-to-equator temperature gradients (ΔSST_N-S_) and Hadley circulation (*12*). Early records from this site used the terrigenous sediment fraction as a proxy for Saharan dust export and documented increased dustiness and the strengthening of 41-kyr (obliquity) dust cycles coincident with iNHG (*13*). This was believed to reflect Saharan aridification in response to high-latitude (glacial-interglacial) climate processes (*1*, *13*, *14*); recent geochemical analyses of Site 659 sediments that parsed the terrigenous fraction into windblown Saharan dust and African fluvial sediment also revealed the persistence of both 41- and 19- to 23-kyr variability in dust fluxes since at least 11 Ma, with a gradual shift toward higher dust fluxes centered around 2.7 Ma originally interpreted as drying (*1*) but later suggested to be strengthening offshore winds (*11*, *15*). This interpretation has anchored our understanding of the Plio-Pleistocene evolution of the African monsoon, and the coincidence of evidence for northern African aridification and increased climate variability with the appearance of some of the earliest members of the genera *Homo* and *Paranthropus* have fueled research into the climatic influences on hominid evolution [e.g., (*1*, *14*)]. While dust export at Site 659 (~18°N) is year-round, it peaks in the winter (*16*), and thus we continue to lack long, highly resolved records of northern African summer rainfall spanning critical Earth system transitions in the Plio-Pleistocene; Mediterranean sapropels (*17*–*19*) and clay mineral geochemistry (*20*, *21*), indices of summer precipitation in the Nile River catchment region, are primarily dominated by 19- to 23-kyr cyclicity, with minor 41-kyr components (*22*) and no decrease in frequency across the Plio-Pleistocene boundary or iNHG. Together, these suggest variable influences of local insolation versus high-latitude temperature and ice volume on northern African environments (*15*).

Leaf waxes from land plants are exported from northern Africa to the Atlantic by easterly winds, and their hydrogen and carbon isotopic compositions (δ^2^H_wax_ and δ^13^C_wax_ values) record past atmospheric circulation and vegetation. δ^2^H_wax_ values are controlled by both the amount of precipitation and potential for evapotranspiration (*23*, *24*), while δ^13^C_wax_ values are primarily controlled by the proportional inputs from plants using the C_3_ and C_4_ photosynthetic pathways; C_3_ plants dominate in tropical rainforests and Mediterranean forests, while C_4_ plants are more common in savanna grasslands (*25*). Existing suborbitally resolved leaf wax isotope–based precipitation and vegetation reconstructions from Site 659 and the adjacent MD03-2705 cover portions of the Pliocene [4.99 to 3.00 Ma (*26*)] and mid- to late Pleistocene [1.102 to 1.009 (*27*), 0.519 to 0.362 (*2**)*, 0.131 to 0.002 Ma (*23*)] but not the key moments of the Plio-Pleistocene when dust records suggest aridification in the Sahara. Here, we present suborbitally resolved δ^2^H_wax_ and δ^13^C_wax_ data from Site 659 spanning 3.30 to 2.48 Ma (*28*), which document the relationships between Plio-Pleistocene Saharan and Sahelian rainfall, high-latitude processes, atmospheric and oceanic circulation, and insolation forcing.

## RESULTS

**Fig. 2.**
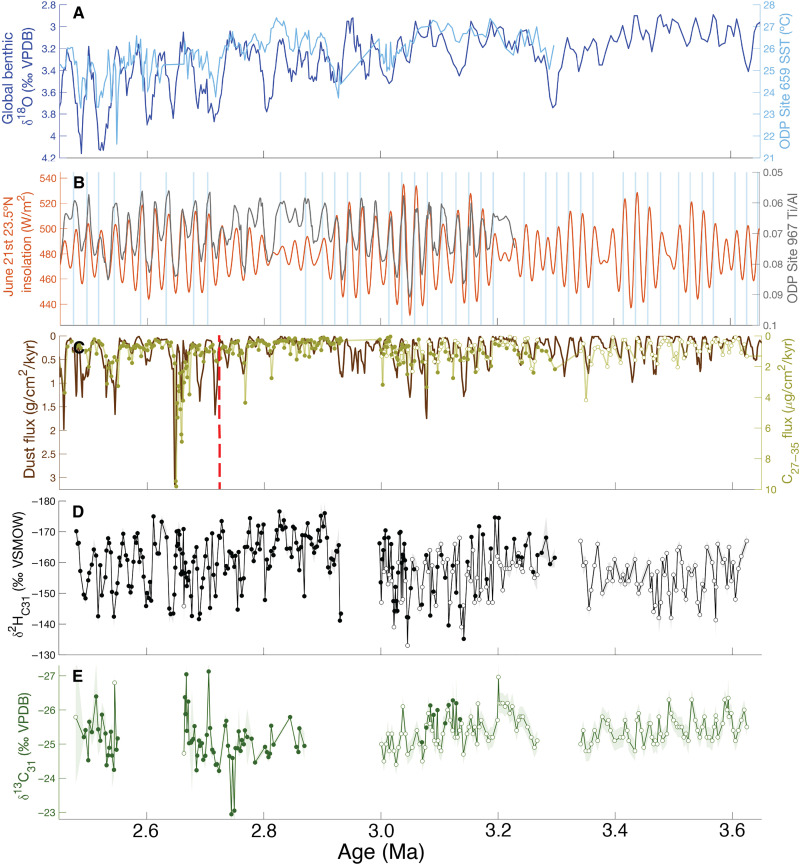
Global and northwestern African climate spanning the Plio-Pleistocene boundary. (**A**) Global benthic foraminiferal δ^18^O stack (*9*) and ODP Site 659 U^k′^_37_-based SSTs (*11*). (**B**) June 21st 23.5°N insolation (*94*) and eastern Mediterranean ODP Site 966 sapropels (blue bars) (*32*) and ODP Site 967 XRF Ti/Al ratios (*20*). (**C**) Site 659 dust (*15*) and C_27–35_ *n*-alkane fluxes. (**D** and **E**) Site 659 δ^2^H_C31_ (D) and δ^13^C_C31_ (E) values. Shading indicates analytical error. Filled circles indicate data generated here, while open circles indicate prior results (*15*, *26*, *50*). Vertical red dashed line indicates the single most statistically significant change in mean dust flux between 11 and 0 Ma; see fig. S1 for full dust flux time series. VPDB, Vienna Pee Dee belemnite.

**Fig. 3.**
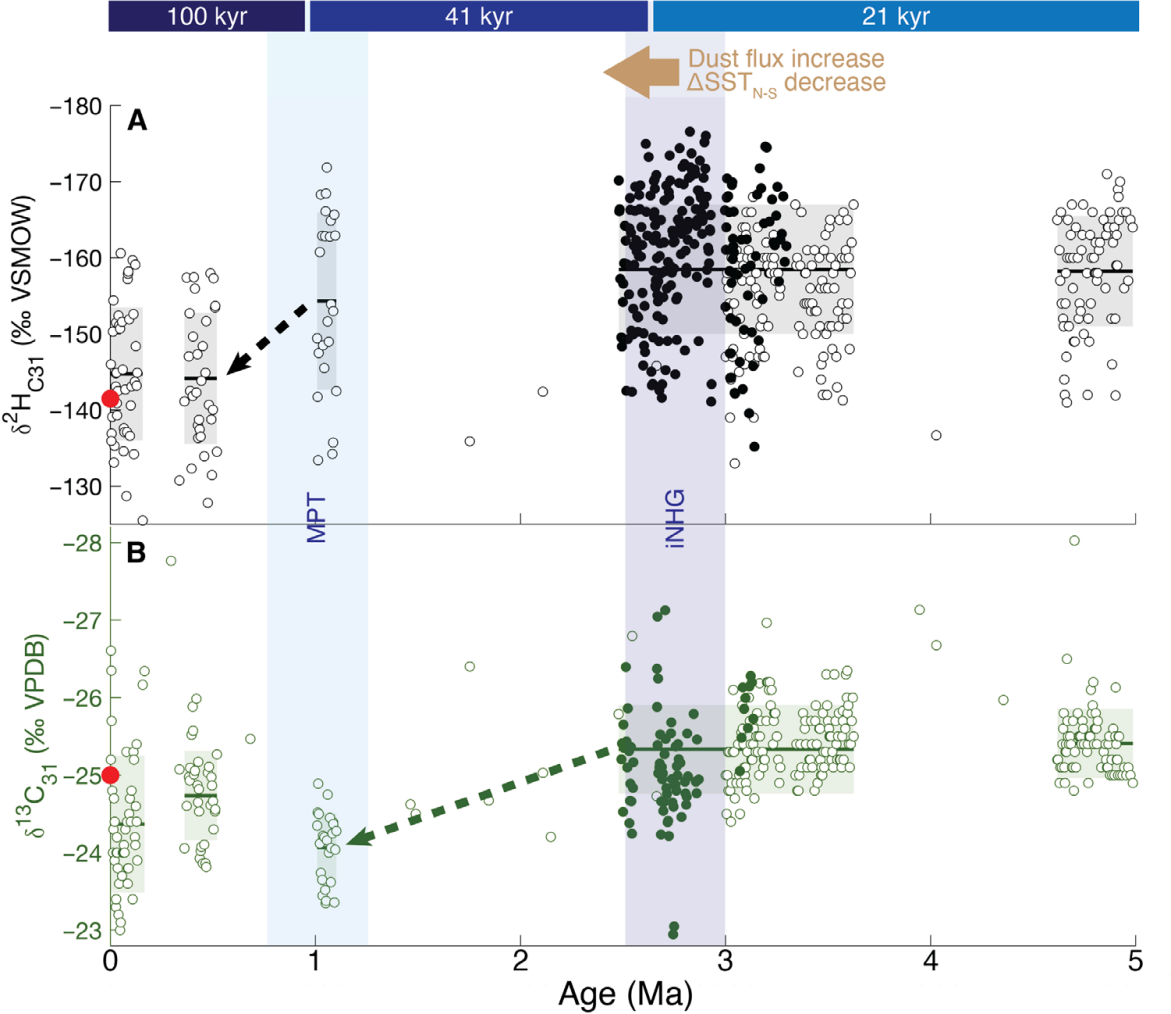
Early Pliocene-present trends in global and northwestern African hydrology and ecology. ODP Site 659 and MD03-2705 (**A**) δ^2^H_C31_ and (**B**) δ^13^C_C31_ compilations with major changes (iNHG and MPT) in glacial-interglacial pacing. Filled circles indicate data generated here, while open circles indicate prior results (*2*, *15*, *23*, *26*, *27*, *50*), 5 to 0 Ma. Lines and shading indicate mean and ±1 σ of bin, respectively. δ^2^H_C31_ values are uncorrected for ice volume. Red circles/lines indicate core-top (i.e., modern) δ^2^H_C31_ and δ^13^C_C31_ values (*23*).

We measured long-chain (C_31_) *n*-alkane δ^2^H and δ^13^C (δ^2^H_C31_, *n* = 229; δ^13^C_C31_, *n* = 80) values spanning 3.30 to 2.48 Ma to establish baseline conditions before, after, and during iNHG (~2.7 Ma). Combined with existing data, our measurements achieve overall resolutions of 3 (δ^2^H_C31_) and 5 kyr (δ^13^C_C31_) from 3.5 to 2.5 Ma but higher within shorter intervals (e.g., 3.7-kyr δ^13^C_C31_ from 2.82 to 2.63 Ma). We observe no major changes in mean δ^2^H_C31_ or δ^13^C_C31_ values from 5 to 2.5 Ma outside of orbital variability (Figs. 2, D and E, and 3, A and B); for example, there is no δ^2^H_wax_ evidence for a 3.8 to 3.3 Ma northern African humid period (*29*).

Between 1 and 0.5 Ma, δ^2^H_C31_ values increase by ~13‰ (Fig. 3A), suggesting that this drying step may have been triggered by major cooling at the mid-Pleistocene transition (MPT) (0.8 Ma). In contrast, δ^13^C_C31_ values reach their modern values (~1‰ enriched relative to the Pliocene; Fig. 3B) by 1 Ma. Regardless, both signify drier and more C_4_-dominated environments in the mid- to late Pleistocene. A wetter (vegetated) northwestern Africa in the Pliocene versus the late Pleistocene is consistent with modeled monsoonal rainfall; in the PlioMIP2 ensemble, Pliocene Sahelian summer (July to October) rainfall was ~300 mm (2.5 mm/day) higher than the present (*30*), equivalent to a 41% increase over the 20th to 21st century mean July to October total (Fig. 1B).

**Fig. 4.**
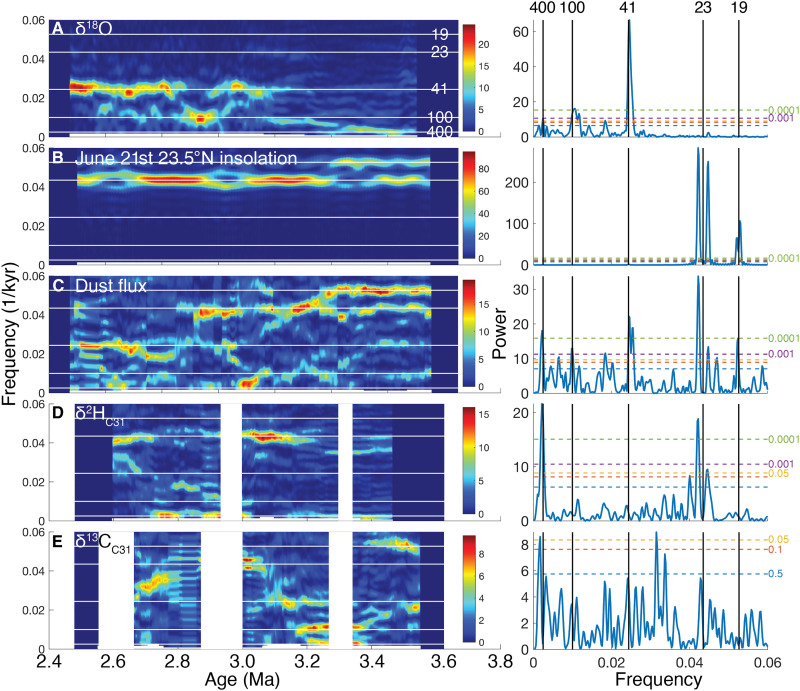
Orbital frequencies in Plio-Pleistocene global and northwestern African climate parameters. Evolutionary (left) and stationary (right) Lomb-Scargle power spectra of the (**A**) global benthic foraminiferal δ^18^O stack (*9*), (**B**) June 21st 23.5°N insolation (*94*), and ODP Site 659 (**C**) dust flux (*15*) and (**D**) δ^2^H_C31_ and (**E**) δ^13^C_C31_ values. Dashed lines indicate false alarm probability, and white boxes indicate core or data gaps.

**Fig. 5.**
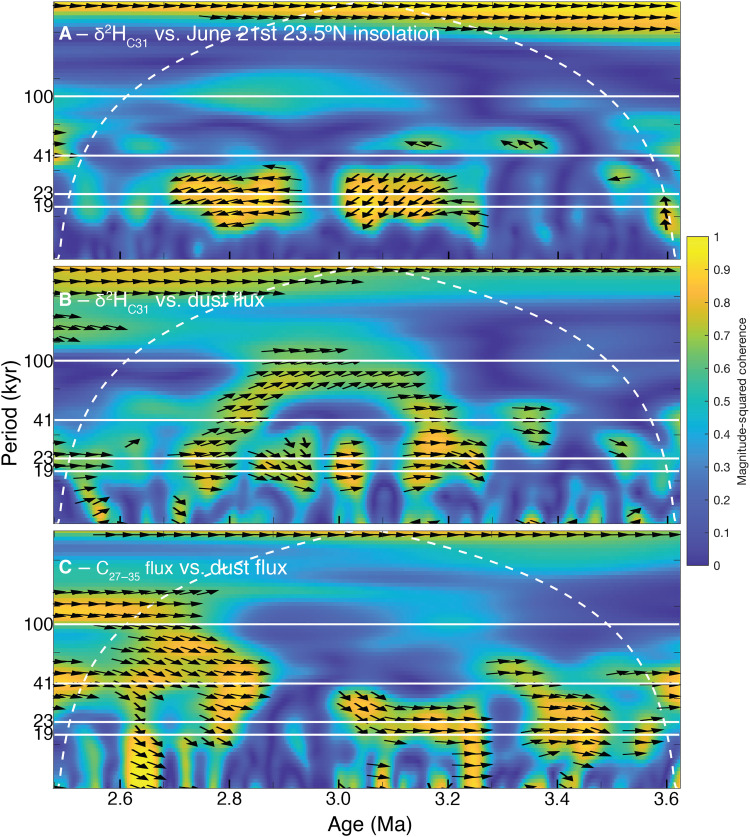
Time-frequency domain correlation between northwestern African paleoenvironmental proxies. Evolutionary wavelet cross-spectra of ODP Site 659 (**A**) δ^2^H_C31_ values and June 21st 23.5°N insolation (*94*), (**B**) δ^2^H_C31_ values and dust flux (*15*), and (**C**) C_27–35_ *n*-alkane and dust fluxes across the Plio-Pleistocene boundary. White dashed line indicates cone of influence. Arrows (≥0.60 magnitude-squared coherence) pointing to the left (A) or right [(B) and (C)] indicate 0° phase lead/lag.

δ^2^H_wax_ shows strong orbital-scale variations over the last 5 Ma with consistent amplitudes of ~30 per mil (‰). From 3.5 to 2.5 Ma, δ^2^H_C31_ values (Figs. 2D and 4D) exhibit pronounced 23- and 400-kyr variability, consistent with eccentricity-modulated precession of summer rainfall. These cycles are spectrally coherent and in phase in the 19- to 23-kyr bands with June 21st insolation at 23.5°N (Fig. 5A). Precessional variability is consistent from the Pliocene to the late Pleistocene, indicating a stable response to insolation forcing with only minor influence from NHG.

In contrast, geochemically based estimates of dust flux vary with both precession and obliquity from at least ~11 Ma (*15*), shifting from predominantly 21- to 41-kyr cycles at about 2.7 Ma, the same time mean dust fluxes increase (Fig. 2C and fig. S1). Dust and *n*-alkane fluxes are highly spectrally coherent and in-phase (Fig. 5C). Their similarity suggests that plant waxes and dust derive from similar regions and *n-*alkane fluxes reflect transport rather than plant growth due to wet conditions. There are no statistically significant (*P* < 0.05) orbital frequencies in the δ^13^C_C31_ data (Fig. 4E).

Before 2.7 Ma, δ^2^H_C31_ values and dust flux are highly spectrally coherent and in phase in the 19- to 23-kyr bands (Fig. 5B). Dust and alkane flux minima are coincident with boreal summer insolation maxima and strengthened summer rainfall (low δ^2^H_C31_ values) (Fig. 5B), suggesting that, during boreal insolation maxima, there was sufficient summer moisture to stabilize the land surface and suppress winter dust export (*31*). After 2.7 Ma, δ^2^H_C31_ and dust/alkane flux cycles are decoupled. Thus, Pleistocene shifts in global climate may have enhanced orbital-scale dust export cycles [e.g., (*1*, *10*, *11*, *15*) and fig. S1] without affecting the magnitude of precipitation cycles, which remain constant around ~30‰ from the Pliocene to present (Figs. 2D and 3A).

## DISCUSSION

### Differing controls on northwestern African summer monsoon rainfall and dust export

June 21st insolation at 23.5°N is highly spectrally coherent with both northeastern African fluvial runoff [via Mediterranean sapropels (*17*–*19*, *32*) and detrital geochemistry (*20*, *21*); Fig. 2B)] and northwestern African summer precipitation (via δ^2^H_C31_ values; Fig. 5A), reinforcing that high boreal summer insolation (during eccentricity-modulated precession minima) enhances the northwestern African land-sea temperature and pressure gradients and therefore the pan-northern African summer monsoon, not just northwestern or northeastern Saharan moisture. [The prominence of the 400-kyr and absence of the 100-kyr eccentricity cycle in the Site 659 δ^2^H_wax_ record merit further study; it appears in other Afro-Mediterranean climate records (*33*–*35*), but there is thus far no consensus mechanism.] The consistency in orbital-scale δ^2^H_C31_ variability and the absence of 41-kyr obliquity cycles across the Plio-Pleistocene transition demonstrates that the strengthening of glacial-interglacial cycles at iNHG had a little effect on the pacing of northwestern African rainfall variations. Nevertheless, the simultaneous onsets of more dust (Fig. 2C and fig. S1), enhanced 41-kyr variability in dust and *n*-alkane transport (Fig. 4C), amplified glacial-interglacial cycles (Fig. 2A), increased northeasterly trade wind-derived pollen at Site 659 (*36*), and iNHG (*8*) at ~2.7 Ma suggest that high-latitude cooling increased aeolian dust and alkane export.

Although changes in dust flux have long been interpreted to reflect high-latitude forcing of rainfall [e.g., (*1*)], we observe a more complex, seasonally variable response to iNHG. The decoupling of δ^2^H_C31_ and dust/alkane flux cyclicity is best explained by the differing seasonal and atmospheric controls on δ^2^H_C31_ values and dust. δ^2^H_C31_ values primarily reflect summer precipitation, whereas dust export to Site 659 is highest in winter-spring (*16*). Dust-based elemental weathering proxies could reflect precipitation, which enhances weathering (*15*), erosion by wind, which exposes more unweathered dust as dust export increases (*37*), or latitudinal shifts in dust source from, for example, low-Fe/K Saharan soils to high-Fe/K savannah soils (*27*, *36*, *38*).

**Fig. 6.**
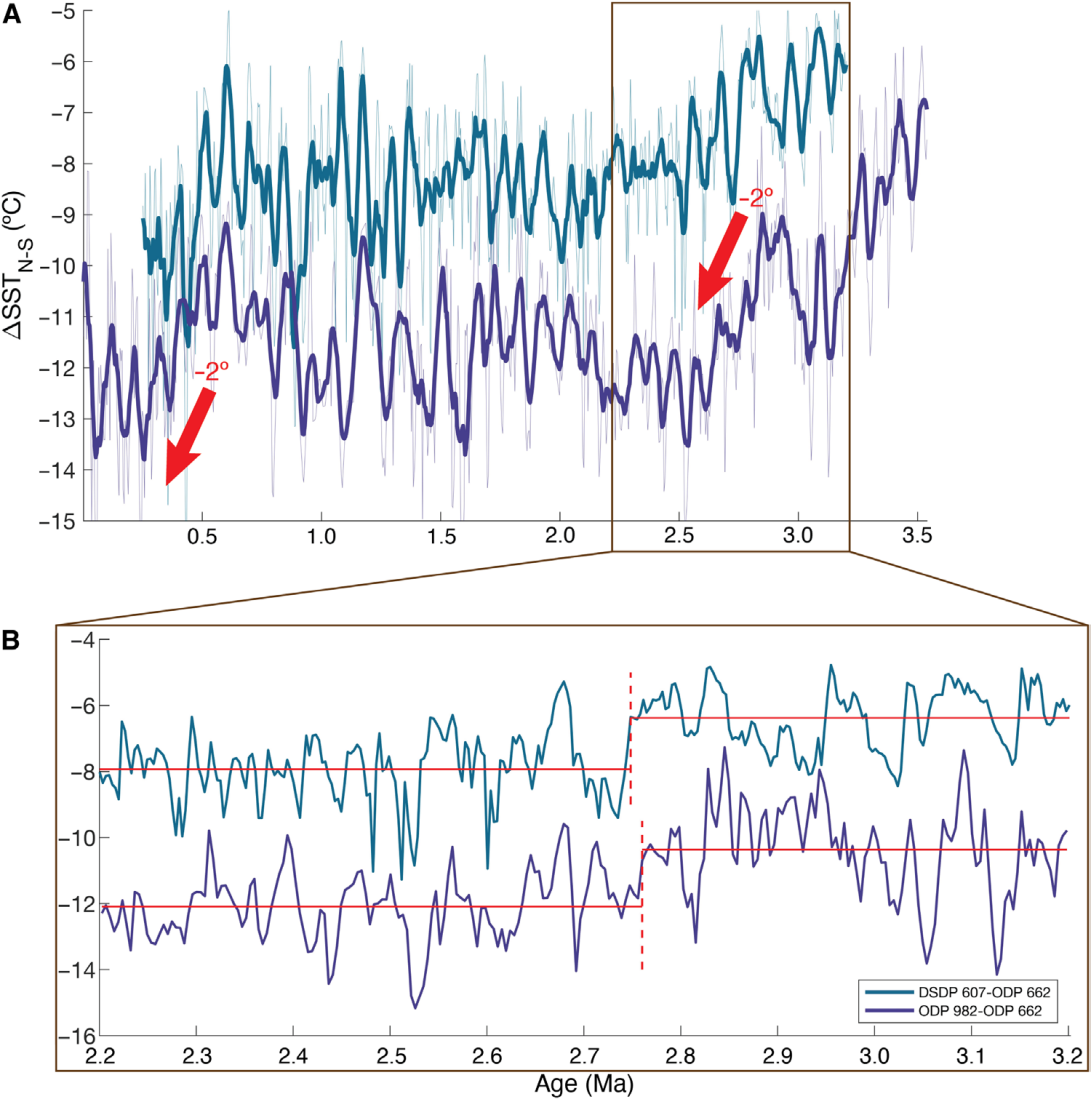
Plio-Pleistocene North Atlantic meridional SST gradients. (**A**) The 3.5 to 0 Ma ΔSST_N-S_ based on U^k′^_37_ values from North Atlantic DSDP Site 607 (*81*) and ODP Site 982 (*82*) and tropical Atlantic ODP Site 662 (*83*) (10-point running average in bold). (**B**) The 3.2 to 2.2 Ma ΔSST_N-S_. Vertical red dashed lines indicate the single most statistically significant change in mean ΔSST_N-S_, and horizontal red lines indicate mean ΔSST_N-S_ before/after this point.

A strengthening and equatorward shift of the mid-latitude westerlies around 2.73 Ma (*10*) suggests that the increase in dust fluxes to ODP Site 659 may be part of a wider atmospheric-oceanic reorganization. A steeper meridional sea surface temperature (SST) gradient—such as the 2°C increase in the North Atlantic at 2.75 Ma (Fig. 6B)—is a possible mechanism for driving stronger trades after iNHG (*15*). A stronger gradient enhances Hadley circulation and therefore the trade winds (*39*–*41*), particularly in the winter due to heightened high-latitude seasonality (*42*). Similarly, polar cooling intensifies the winter Libyan high/anticyclone and thereby the wintertime trades on its periphery (*43*). The synchronicity between enhanced wind strength [dust flux and x-ray fluorescence (XRF) Zr/Rb ratios (*11*, *15*)], high-latitude cooling, and Atlantic ΔSST_N-S_ at 2.7 Ma suggests a close relationship such as that observed and modeled on decadal (*41*, *44*, *45*), glacial-interglacial (*38*, *41*, *46*), and longer (*47*) timescales, including the Pliocene, where reduced meridional and zonal SST gradients weaken lower tropospheric winds (*48*). In contrast, our data show that precipitation over this interval was dominated by variations in tropical boreal summer insolation and their impacts on the summer monsoon.

In short, different aspects of atmospheric circulation control precipitation versus dust export, and so the decoupling of summer monsoon-driven rainfall and trade wind strength allows for divergent spectral evolution of δ^2^H_C31_ and dust fluxes. This underscores that dust fluxes are not solely moisture indices and implies asymmetrical future responses of summer monsoonal precipitation and wintertime dust export to anthropogenic warming.

### Plio-Pleistocene evolution of northwestern African hydrology

Mid- to late Pleistocene (0.53 to 0.35 and 0.13 to 0 Ma) δ^2^H_C31_ values not only retain a ~21-kyr imprint but also exhibit 41-kyr (obliquity) cyclicity, attributed to the influence of the cross-equatorial (23.5°N to 23.5°S) insolation gradient (*2*, *23*). Unfortunately, these records are short (<158 kyr) and/or of low temporal resolution [~5 kyr (*2*)], spanning two or fewer obliquity cycles, and only the 19- to 23-kyr component is statistically significant above the 95% false alarm probability (0.53 to 0.35 Ma) (fig. S2). Thus, one possibility is that 41-kyr cyclicity in the latest Pleistocene precipitation is a statistical artifact, a single failed precession cycle, etc. If the 41-kyr variability is robust, however, our data suggest that it is unrelated to the cross-equatorial insolation gradient, which should be stable through time.

A second possibility is that the relationship between summer monsoon intensity and insolation forcing has evolved over time—in other words, the relative impacts of insolation versus other forcings—greenhouse gases (GHGs), SSTs, etc.—on precipitation have evolved. Climate models suggest that Sahelian precipitation was ~60% orbitally forced and 40% GHG forced during the last deglaciation (*49*). The orbitally forced (i.e., 21-kyr insolation) component may have been greater during the Pliocene than during the higher-amplitude glacial-interglacial “41-” and “100-kyr worlds” of the mid- to late Pleistocene.

Last, a third possibility is that 41-kyr δ^2^H_C31_ variability is only induced once ΔSST_N-S_ crosses a certain threshold—the drop in ΔSST_N-S_ at 2.75 Ma is only about 2°C (Fig. 6B) (*15*). It declines by an additional ~2°C near 0.45 Ma (Fig. 6A), about when the first δ^2^H_C31_ 41-kyr cycle is documented (fig. S2A). These arguments are speculative, however, and additional higher-resolution data would better constrain possible obliquity variations in the late Pleistocene and shed light on these hypotheses.

### Controls on Plio-Pleistocene northwestern African landscape ecology

Widespread C_4_ expansion occurred across northwestern Africa from 10 to 5 Ma, when δ^13^C_C31_ values at Site 659 increased by ~5.5‰ (+ 37% C_4_) (*15*, *50*). In contrast, from 5 to 0 Ma, δ^13^C_C31_ values are relatively stable, with only a ~1‰ (+ ~7% C_4_) increase from the late Pliocene (3.62 to 2.48 Ma) to the late Pleistocene (0.15 to 0 Ma) (Fig. 3B). The primary influences on C_3_/C_4_ abundance include temperature, rainfall, and *p*CO_2_ (*51*), and of these, precipitation is unlikely to be the dominant control given the lack of correlation between δ^2^H_C31_ and δ^13^C_C31_ values on 10^4^-year (Figs. 2 and 4) to 10^6^-year (*50*) timescales. In addition, the 2.48 to 0.53 Ma drying implied by δ^2^H_C31_ values and models did not induce major changes in C_4_ abundance (Fig. 3).

Changing atmospheric *p*CO_2_ is commonly invoked to explain changing C_3_/C_4_ abundances, particularly that associated with Miocene grassland expansion (*50*), as the C_4_ pathway is generally favored at lower *p*CO_2_. In the late Pleistocene, δ^13^C_C31_ values vary with *p*CO_2_ at a rate of ~2 to 2.5‰ per ~100 parts per million (ppm) (*2*); however, the 2.5 to 0.5 Ma δ^13^C_C31_ increase is only ~0.5‰ per 120 ppm assuming Pliocene and preindustrial values of ~400 (*52*) and 280 ppm (*53*), respectively. Reconciling these observations is challenging. One possibility is that drying dampens the effect of *p*CO_2_: higher precipitation expands the savanna belt (15° to 20°N) (+ C_4_; + δ^13^C_C31_) (*2*, *23*), while drying contracts the Sahel (− C_4_; − δ^13^C_C31_), partially counteracting declining *p*CO_2_ (+ C_4_; + δ^13^C_C31_). Ultimately, deconvolving the influences of *p*CO_2_, seasonality, temperature, and rainfall using solely δ^13^C_wax_ values is unrealistic, although the sign of the Plio-Pleistocene δ^13^C_C31_ change is consistent with declining *p*CO_2_.

### Future directions

Our high-resolution leaf wax isotope record demonstrates remarkably consistent northwestern African summer monsoonal rainfall behavior across the Plio-Pleistocene boundary despite global cooling, climate events such as iNHG, and intensified 41-kyr glacial-interglacial cycles, standing in stark contrast to the body of dust flux records traditionally interpreted as a hydroclimate index. Instead, the combination of biomarker isotope and sedimentological proxies reveals differing seasonal responses of northwestern African atmospheric circulation to global conditions. The heightened 41-kyr variability in northern African dustiness, coincident with higher mean dustiness, global trade wind intensification, and a steepened North Atlantic meridional SST gradient, demonstrates that northwestern African winter climate is highly sensitive to distal, high-latitude forcings. In contrast, the summer monsoon marched to the beat of low-latitude summertime insolation. In this context, the ^2^H enrichment, signifying drying, that we observe between the early and late Pleistocene (Fig. 3A) remains an unsolved challenge.

Available Site 659 data now suggest that baseline precipitation intensity remains at Pliocene levels until the MPT, after the landscape has already shifted toward more C_4_ plants, but the large gaps between 2.5 to 1 and 1 to 0.5 Ma obscure the northern African climate response to state changes such as emergence of zonal SST gradients in the tropics, strengthened pole-to-equator temperature gradients, and the intensification of 100-kyr glacial cycles, all of which may have affected the African summer monsoon. Additional data from these intervals will document the Pleistocene history of the African summer monsoon and improve our understanding of the environmental context for hominin evolution and migration out of Africa.

## MATERIALS AND METHODS

### Experimental design

Past biomarker (isotope)-based paleoenvironmental studies using Site 659 material have focused on the earlier Pliocene and later Pleistocene, but key climate transitions such as the iNHG or MPT remain undercharacterized. To better understand how the Plio-Pleistocene transition (2.58 Ma) and late Pliocene global cooling (~2.7 Ma) may have altered the frequency and severity of northwestern African monsoon cycles, we targeted the 3.5 to 2.5 Ma interval, sampling at 3- to 5-kyr resolution to resolve precessional variability.

### Northwestern African climatic-geologic setting

Modern northwestern African rainfall is highly seasonal, the result of meridional migration of the tropical rain belt (TRB) between ~10° (winter) and 20°N (summer) (*54*). At the Sahara-Sahel boundary, near the latitude of Site 659, annual precipitation is heavily weighted toward the summer months (Fig. 1A). Northwestern African precipitation δ^2^H (δ^2^H_H2O_) and δ^18^O (δ^18^O_H2O_) values are strongly anticorrelated with amounts across time and space—seasonal monsoonal intensification and individual storm events are characterized by ^18^O depletion (*55*, *56*), and 20th century monthly average δ^2^H_H2O_ values and amounts are strongly anticorrelated [coefficient of determination (*R*^2^) = 0.86] at Bamako, Mali, the northwestern African Global Network of Isotopes in Precipitation (GNIP) station with the highest historical data density (*57*), which has an extreme seasonal rainfall cycle (Fig. 1B). Modern coastal Northwestern African core top δ^2^H_wax_-inferred δ^2^H_H2O_ values also follow spatial patterns in rainfall amount (*R*^2^ = 0.52) (*58*). Interannually, summer δ^18^O_H2O_ values track Sahelian precipitation amount (*59*), and on even longer (glacial-interglacial) timescales, low δ^2^H_wax_-inferred δ^2^H_H2O_ values correspond to high lakestands throughout northern and central Africa (*60*) and known intervals of a wetter Sahara such as the African humid period (~0.015 to 0.005 Ma) (*7*, *23*, *58*, *60*–*62*). The latitudinal vegetation distribution is set by the TRB, with Sudanian woodlands and Sahelian savanna dominating at 15° to 20°N and desert at 20° to 30°N (*63*).

Offshore dust has been sourced from the interior Sahara since at least 11 Ma, as indicated by the radiogenic isotopic signature of the lithogenic fraction at Site 659 (*15*, *64*). Saharan dust is transported offshore year-round by the low-altitude northeasterly trade winds, supplemented by the higher-altitude Saharan Air Layer in boreal summer. However, at the latitude of Site 659 (18°N), the export of terrigenous material is dominated by the Harmattan-driven winter western African dust plume, while summer storms follow more northerly trajectories and deposit further west. Thus, specifically for Site 659, dust deposition records wintertime atmospheric circulation (*16*, *65*, *66*). Further evidence suggests that the trades intensify during Northern Hemisphere glacials (*67*). Therefore, Site 659 dust levels have a strong sensitivity to wintertime northerly latitude climate and transport capability (i.e., wind strength and gustiness), while δ^2^H_wax_ values reflect summer rainfall intensity due to extremely dry winters (Fig. 1B) and wax production biased toward the (summertime) growing season (*24*).

### Leaf wax biomarker isotope proxies

Plant epicuticular waxes form protective coatings on leaves (*68*). Longer-carbon chain (>C_24_) *n*-alkanes are primarily derived from higher-order terrestrial plants and grasses and are well preserved in marine sediment. Sedimentary *n*-alkane δ^2^H_wax_ and δ^13^C_wax_ values integrate climate and vegetation signals across broad regions (10^2^ to 10^4^ km^2^) rather than localized (<10 km^2^), basin-scale processes (*24*). Assuming sediment and waxes are exported by the same mechanisms, modern northwestern African marginal waxes are primarily derived from major dust storms (*69*). While past humid periods activated extensive river systems and wadi floods, increasing fluvial sedimentation (*70*), the high spectral coherence between dust and alkane fluxes (Fig. 5C)—and not moisture (i.e., δ^2^H_wax_ values)—suggests that wind has been the primary pathway, else we might observe a stronger relationship between δ^2^H_wax_ values and alkane fluxes.

δ^2^H_wax_ and δ^2^H_H2O_ values are highly correlated, with lower values indicating wetter conditions (*24*). Given the stability of the seasonal temperature cycle, constancy of northwestern African topography over the Plio-Pleistocene and proximity to the warm, moisture-generating tropical Atlantic, temperature, altitude, and source effects here are thought to be minimal (*23*), and we interpret tropical northern African δ^2^H_H2O_ values as largely reflective of rainfall amount (*71*, *72*).

δ^13^C_wax_ values primarily record plant photosynthetic pathway (C_3_/C_4_/CAM), which is determined by water availability, *p*CO_2_, temperature, and other ecological processes (*51*, *73*, *74*), with high values indicating predominantly C_4_ over C_3_ plants. CAM vegetation in northern Africa is negligible (*63*), and offshore northwestern African core top δ^13^C_wax_ values closely resemble the latitudinal distribution of C_3_/C_4_ vegetation (*2*, *75*). We estimate the quantitative proportion of C_3_/C_4_ vegetation based on tropical C_3_/C_4_ endmember values of −36.7 ± 3.2‰ and −22.0 ± 2.6‰, respectively (*76*).

As a further check on the capabilities of δ^2^H_wax_ values as recorders of hydrology, we calculate present-day δ^2^H_H2O_ values (*61*) using core top δ^2^H_C31_ values (*23*), δ^13^C_wax_ value–based C_3_/C_4_ estimates, and alkane-specific δ^2^H_wax_ δ^2^H_H2O_-δ^2^H_C31_ fractionation factors from a modern tropical plant dataset (*76*). Core top δ^2^H_C31_ and δ^13^C_C31_ values yield a late Holocene δ^2^H_H2O_ value within 15‰ of modern amount-weighted annual mean δ^2^H precipitation values (Fig. 1B), suggesting that our wax-based water isotope reconstructions are realistic in this setting.

### Leaf wax *n*-alkane extraction, purification, and quantification and δ^2^H_wax_ and δ^13^C_wax_ value measurement

We extracted lipids from freeze-dried, homogenized sediment using a Thermo Fisher Scientific (Dionex) Accelerated Solvent Extractor 350 (solvent: 9:1 dichloromethane:methanol) and separated and purified *n*-alkanes via sequential aminopropyl and silica gel flash column chromatography. *N*-alkane abundances and concentrations were quantified using Agilent 6890 and 7890 gas chromatography (GC)–flame ionization detectors with Agilent HP-1ms columns (30 m by 0.25 mm by 0.25 μm) and the TEXPRESS MATLAB package (*77*).

An Agilent 6890 GC with an RTX-5 MS column (30 m by 0.32 mm by 0.25 μm) coupled to a Thermo Delta V Plus isotope ratio mass spectrometer (IRMS) [pyrolysis (hydrogen) reactor held at 1410° to 1425°C; combustion (carbon) reactor at 1100°C] was used to measure *n*-alkane δ^2^H and δ^13^C values. GC oven temperatures were held for 2 min at 50°C, increased to 230°C at 15°C/min, increased to 320°C at 4°C/min, and held for 8 min. H_3_^+^ factors (*78*) were measured every 2 to 3 days, averaging 1.85 ± 0.05 (1 σ). An *n*-alkane standard mixture (δ^2^H_C29_ = −162.6 ± 2.2‰ Vienna standard mean ocean water; δ^2^H_C31_ = −271.9 ± 2.0‰; δ^2^H_C32_ = −212.4 ± 1.0‰; δ^13^C_C29_ = −29.30 ± 0.02‰ Vienna Pee Dee belemnite; δ^13^C_C31_ = −29.43 ± 0.01‰; δ^13^C_C32_ = −29.47 ± 0.02‰) from Arndt Schimmelmann (Indiana University) was injected between every two samples, and the difference between measured and reported δ^2^H_C29–32_ and δ^13^C_C29–32_ values was used to correct for IRMS offset and drift. Reported δ^2^H_wax_ and δ^13^C_wax_ errors are ±1 σ if *n* = 3, the difference between duplicates if *n* = 2, and the difference between preceding/subsequent standard δ^2^H_C31_ and δ^13^C_C31_ values if *n* = 1.

Samples were generally rich in long-chain *n*-alkanes, with the C_31_ homolog typically the most abundant. δ^2^H_C31_ values in our samples are highly correlated with δ^2^H_C29_ (*R*^2^ = 0.70) and δ^2^H_C33_ values (*R*^2^ = 0.62); δ^13^C_C31_ are correlated with δ^13^C_C29_ (*R*^2^ = 0.70) and δ^13^C_C33_ (*R*^2^ = 0.48) values (fig. S3). Therefore, interpretations are based on the δ^2^H_C31_ and δ^13^C_C31_ records.

### Carbon preference index

The carbon preference index (CPI), the ratio of odd- to even-carbon chain length alkanes, indicates degree of diagenetic degradation (*79*, *80*), where highly altered hydrocarbons’ CPI ≈ 1.

$${\text{CPI}}_{27–35}=\left[\Sigma_{\text{odd}}\left(C_{27}–C_{33}\right)+\Sigma_{\text{odd}}\left(C_{29}–C_{35}\right)\right]/2\left[\Sigma_{\text{even}}\left(C_{28}–C_{34}\right)\right]$$

CPI_27–35_ averages 5.50 between 4.99 and 2.28 Ma, indicating good alkane preservation. (Three samples with CPI_27–35_ values below 1.5 were excluded from analyses.)

### Existing data and age model

By combining later Plio-Pleistocene (3.30 to 2.48 Ma) with existing earlier Pliocene (4.99 to 3.00 Ma) measurements (*15*, *26*, *50*), we achieved δ^2^H_C31_ and δ^13^C_C31_ resolutions of 3.2 and 5.3 kyr, respectively, from 3.62 to 2.48 Ma. All results and prior data are plotted on the most recent age model (*11*, *15*), which is based on benthic oxygen isotope stratigraphy and tuning of ln (Ca/Fe) values to high-latitude boreal summer insolation. The δ^2^H_wax_ and δ^13^C_wax_ data gap between 2.998 and 2.932 Ma is due to clear coring disturbances that would have compromised data quality in those sections (*11*).

### SST gradient and change point calculations

We used U^k′^_37_-based SST records from the North [Deep Sea Drilling Program (DSDP) Site 607 (*81*) and ODP Site 982 (*82*)] and equatorial Atlantic [ODP Site 662 (*83*)] to derive the Atlantic pole-to-equator temperature gradient (ΔSST_N-S_) across the Plio-Pleistocene boundary.

The most statistically significant changes in mean ΔSST_N-S_ between 3.2 (the start of the DSDP Site 607 U^k′^_37_ record) and 2.2 Ma and in the dust flux between 11 and 0 Ma were identified using MATLAB’s findchangepts function (*84*, *85*). Both ΔSST_N-S_ we calculated (DSDP Site 607–ODP Site 662 and ODP Sites 982 and 662) exhibit a ~2°C decrease around 2.75 Ma (Fig. 6B), coincident with the dust flux change point and the beginning of its strengthened 41-kyr periodicity.

### Statistical analysis

ΔSST_N-S_ calculation methodology is described in (*86*); briefly, northern and equatorial data were interpolated to the same constant time step, the mean resolution of the lower-resolution record. Mean-based change points in ΔSST_N-S_ and dust flux data were identified using MATLAB’s findchangepts function (*84*, *85*). We used stationary and evolutionary Lomb-Scargle power spectra (MATLAB’s plomb function) (*87*, *88*) to characterize frequencies in unevenly sampled time series data and avoid introducing bias via constant time step resampling. We used magnitude-squared wavelet coherence (MATLAB’s wcoherence function) (*89*–*91*) to assess the evolutionary correlation between two time series in the time-frequency plane after resampling both to the mean resolution of the lower-resolution record.
