# Supplementary material for: Fundamentally unchanged northwestern African rainfall regimes across the Plio-Pleistocene transition
Source: Sci Adv. 2025 Jun 20;11(25):eads3149. doi: 10.1126/sciadv.ads3149 (PMC12180497; doi:10.1126/sciadv.ads3149)
Supplement: Supplementary file 1 — Figs. S1 to S3 Legend for table S1 [file sciadv.ads3149_sm.pdf]

Science Advances
AAAS

Supplementary Materials for

Fundamentally unchanged northwestern African rainfall regimes across the Plio-Pleistocene transition

Bryce A. Mitsunaga *et al.*

Corresponding author: Bryce A. Mitsunaga, bryce_mitsunaga@g.harvard.edu

Sci. Adv. **11**, eads3149 (2025)
DOI: 10.1126/sciadv.ads3149

The PDF file includes:

Figs. S1 to S3
Legend for table S1

Other Supplementary Material for this manuscript includes the following:

Table S1

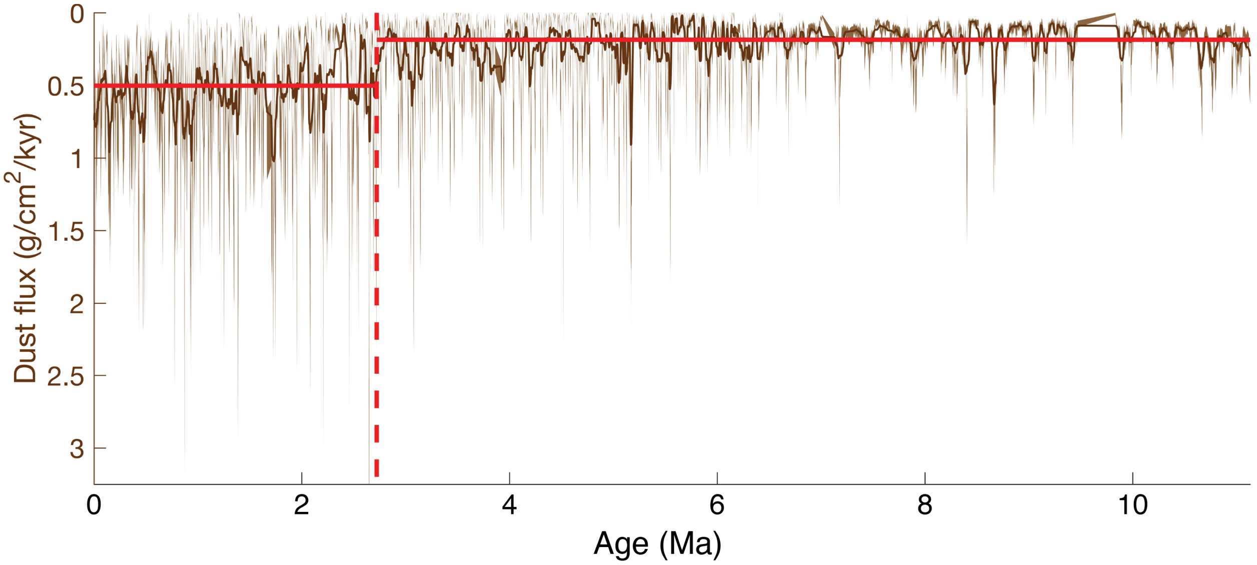

Fig. S1. Northwestern African dust export since the mid-Miocene. Twenty-five-point running average of ODP Site 659 dust flux values with 5th-95th percentiles shaded (*15*). Vertical red dashed line indicates the single most statistically significant change in mean dust flux and horizontal red lines indicate mean values before / after this point (2.72 Ma).

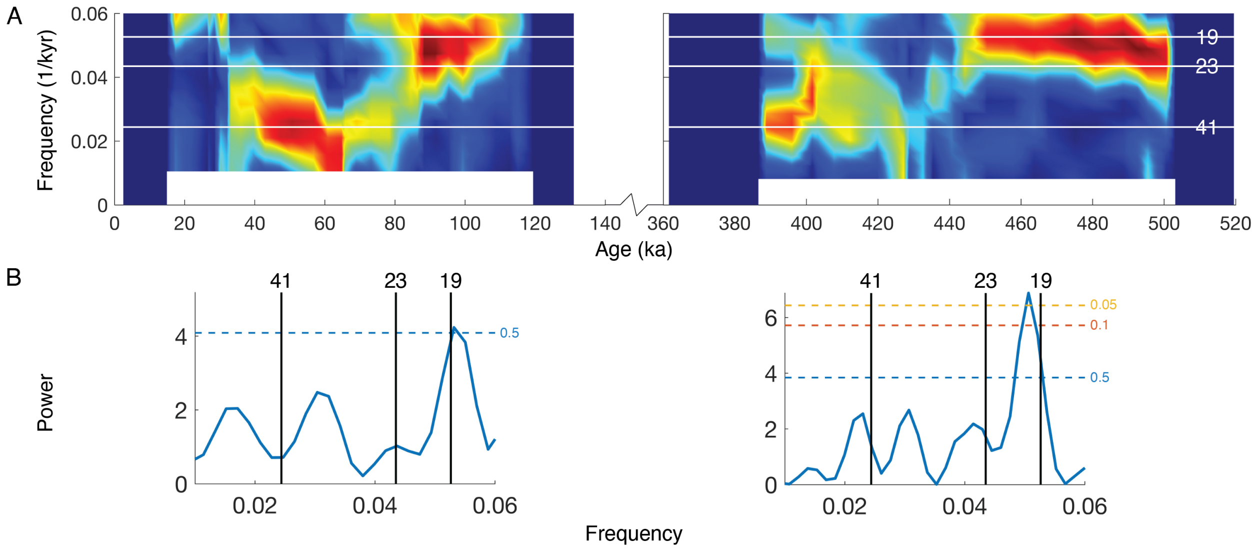

Fig. S2. Orbital frequencies in later Pleistocene northwestern African hydrology. (**A**) Evolutionary and (**B**) stationary Lomb-Scargle periodograms of ice volume-corrected Pleistocene δ^2H_{C31} values from ODP Site 659 (*23*) and MD03-2705 (*2*). Dashed lines indicate false alarm probability.

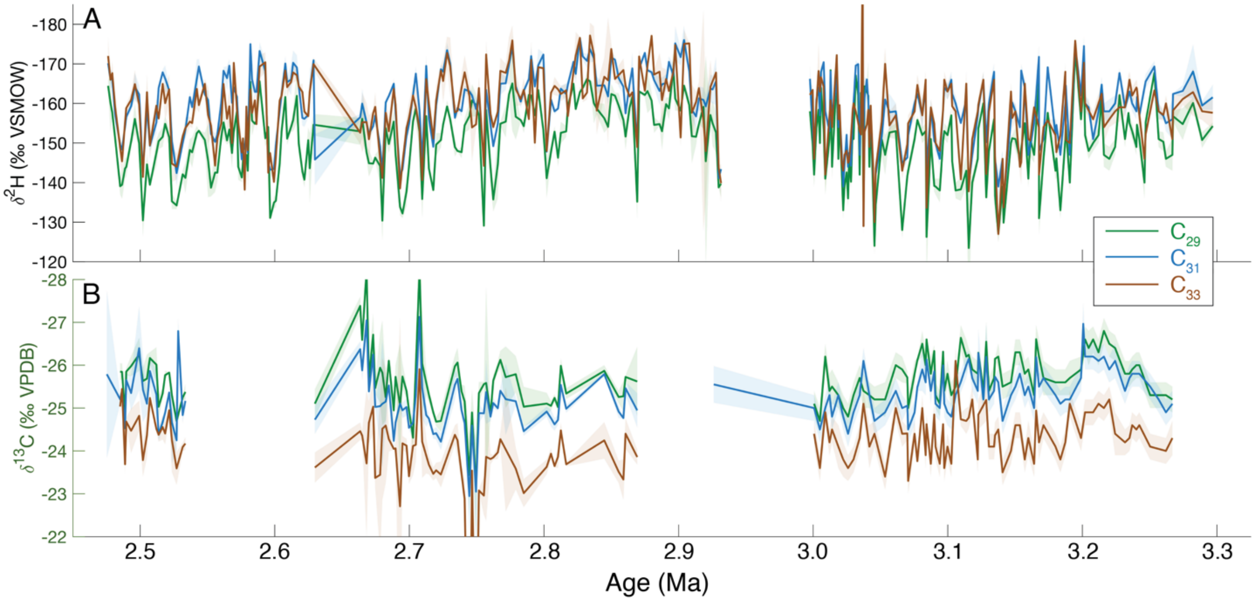

Fig. S3. *N*-alkane δ^2H_{wax} and $\delta^{13}C_{wax}$ values by chain length. (**A**) ODP Site 659 δ^2H_{C29}, δ^2H_{C31}, δ^2H_{C33}, (**B**) $\delta^{13}C_{C29}$, $\delta^{13}C_{C31}$, and $\delta^{13}C_{C33}$ values from 3.3 to 2.45 Ma, including all data generated for this study. For a similar comparison from 5-3.3 Ma, see (*26*).

Table S1. δ^2H_{wax}, $\delta^{13}C_{wax}$, and C_{27-35} *n*-alkane ratios and abundance data.
